# Pharmacological Targeting of Bcl-2 Induces Caspase 3-Mediated Cleavage of HDAC6 and Regulates the Autophagy Process in Colorectal Cancer

**DOI:** 10.3390/ijms24076662

**Published:** 2023-04-03

**Authors:** Donglin Yang, Liujun He, Shuiqing Ma, Shiqiang Li, Yajun Zhang, Chunsheng Hu, Jiuhong Huang, Zhigang Xu, Dianyong Tang, Zhongzhu Chen

**Affiliations:** 1 College of Pharmacy, National & Local Joint Engineering Research Center of Targeted and Innovative Therapeutics, Chongqing Key Laboratory of Kinase Modulators as Innovative Medicine, Chongqing University of Arts and Sciences, Chongqing 402160, China; 2 College of Pharmaceutical Sciences and Chinese Medicine, Southwest University, Chongqing 400715, China

**Keywords:** compound **6d**, colorectal cancer cells, Bcl-2, caspase 3, autophagy, HDAC6

## Abstract

Compound **6d**, a spiroindoline compound, exhibits antiproliferative capability against cancer cell lines. However, the exact underlying mechanism of this compound-mediated inhibitory capability remains unclear. Here, we showed that compound **6d** is an inhibitor of Bcl-2, which suppresses CRC growth by inducing caspase 3-mediated intrinsic apoptosis of mitochondria. Regarding the underlying mechanism, we identified HDAC6 as a direct substrate for caspase 3, and caspase 3 activation induced by compound **6d** directly cleaves HDAC6 into two fragments. Moreover, the cleavage site was located at D1088 in the DMAD-S motif HDAC6. Apoptosis stimulated by compound **6d** promoted autophagy initiation by inhibiting interaction between Bcl-2 and Beclin 1, while it led to the accumulation of ubiquitinated proteins and the reduction of autophagic flux. Collectively, our findings reveal that the Bcl-2-caspase 3-HDAC6 cascade is a crucial regulatory pathway of autophagy and identify compound **6d** as a novel lead compound for disrupting the balance between apoptosis and autophagy.

## 1. Introduction

Apoptosis, a physiological form of cell death, is mediated by two major pathways: the death receptor-induced extrinsic pathway and the mitochondria-mediated intrinsic pathway [1]. Abnormal cell survival through resistance to apoptosis is considered a primary defense mechanism against tumorigenesis and plays a key role in chemoresistance [2]. Mitochondria-mediated apoptosis is fundamentally orchestrated by B-cell lymphoma 2 (Bcl-2) family proteins, which are central regulators and consist of pro-apoptotic proteins, such as Bax (Bcl-2-associated X protein), Bak (BCL-2 antagonist killer 1), Bim (Bcl-2-interacting mediator), Bid (BH3-interacting domain), and Puma (p53 upregulated modulator of apoptosis), and anti-apoptotic proteins such as Bcl-2, Bcl-XL, Bcl-W, Bcl-2-A1, and MCL1 (Myeloid cell leukemia 1) [3,4]. The balance between pro-apoptotic BH3 (Bcl-2 homology 3) only proteins and anti-apoptotic proteins determines whether a cell will survive or undergo apoptosis [5]. Bcl-2 and other related anti-apoptotic proteins directly bind and sequester BH3-only proteins, and then activate the central effectors of apoptosis, Bax and Bak. Subsequently, active Bax and Bak undergo large conformational changes and oligomerization, resulting in the formation of a pore in the mitochondrial membrane and triggering mitochondrial outer membrane permeabilization (MOMP) [6,7]. The formation of MOMP initiates the release of cytochrome c from the mitochondrial intermembrane space into the cytoplasm, which in turn activates the critical caspase cascade and the generation of the apotosome [8,9]. This imbalance between pro- and anti-apoptotic processes is thought to be deregulated in many malignancies, including hematologic malignancies and solid tumors, and leads to chemoresistance [10,11,12,13,14]. Since the physiological level of Bcl-2 in normal dividing cells is low, it is considered an ideal target for cancer therapy. However, some of the reported small molecule inhibitors, which were originally designed to target Bcl-2, have shown acquired resistance due to mutations in the BH3-binding pocket of the Bcl-2 protein, overexpression, and increased oncogenic dependence on alternative antiapoptotic proteins [15,16]. Therefore, the development of new inhibitors, which specifically target Bcl-2 and have completely different chemical structures compared to the reported ones, is urgently needed to offer more effective treatment in patients with colorectal tumors and other types of tumors.

Autophagy plays a role in sustaining cancer cell survival during conditions such as oxide and metabolic stresses, and might mediate tolerance to some treatments such as chemotherapy, radiation and targeted drugs used in patients [17]. It is made up of two basic processes: activation of autophagy, so-called formation of autophagosomes, and fusion of autophagosome and lysosome to form autophagolysosomes where the components are degraded [18]. A previous study has reported that anti-apoptotic proteins from the Bcl-2 family play a vital role not only in the suppression of apoptosis but in the inhibition of autophagy initiation [19]. Bcl-2 and Bcl-xL bind to Beclin 1 directly through inserting into the hydrophobic groove of its BH3 domain to inhibit autophagy, and the dissociation of these proteins from Beclin 1 is essential to autophagy activation in response to stress, both in cultured cells and in vivo [20]. However, to our knowledge, the underlying molecular link between Bcl-2 and the fusion of autophagosome and lysosome has not been reported. Autophagy is involved in the transfer and disposal of cytoplasmic cargo, sequestering misfolded proteins, macromolecular complexes, and organelles [17,21]. The deficiency of autophagic flux results in the accumulation of cytotoxic misfolded protein aggregates that induce cell death [22,23]. Histone deacetylases (HDACs) are a family of enzymes that function in the acetylation of histones and the remodeling of chromatin [24]. Increasing studies have recognized them as therapeutic targets in cancer. Interestingly, only HDAC6 has intrinsic ubiquitin binding capability and is associated with both the microtubules and the F-actin cytoskeleton. HDAC6 plays a vital role in both the cellular clearance of protein aggregates and the autophagosome-lysosome fusion in the autophagy process [23,25]. Therefore, the development of novel inhibitors targeting molecules associated with the autophagy process has been regarded as a therapeutic approach.

Natural and synthetic spiroindolines with indole derivatives are an important class of heterocyclic compounds that exert a wide range of biological properties, such as anticancer and insecticidal activities [26]. In our previous report, a diverse suite of spiroindolines were synthesized using a metal-free, one-pot post-Ugi/diastereoselective domino cyclization in acidic conditions under microwave irradiation, and it was found that compound **6d** exhibits antiproliferative capability against several types of cancer cell lines [27]. However, the underlying mechanism of this compound-mediated inhibitory capability in cancer cells, the potential target, and the effect on colorectal cancer (CRC) cells remain unclear.

In this study, we aimed to explore the anti-tumor activity of Compound **6d** against CRC in vivo/vitro and to elucidate the underlying molecular mechanism. We found that compound **6d** reduces the proliferation and growth of CRC in vitro/vivo and is a novel potential inhibitor targeting Bcl-2. Pharmacological-inhibiting Bcl-2 promoted mitochondrial-dependent apoptosis, activation of the final apoptotic executor, caspase 3, and autophagy initiation, whereas it blocked the fusion of autophagosome and lysosome. Interestingly, we identified that HDAC6 is a substrate of caspase 3 and compound **6d** induces HDAC6 cleavage by activating caspase 3. Moreover, the aspartic acid 1088 (D1088), located in the DMAD-S motif at the C-terminus of HDAC6, was the actual site of proteolytic cleavage by caspase 3 in the presence of compound **6d**, thereby resulting in the accumulation of ubiquitinated protein aggregates. These findings identify a novel inhibitor of Bcl-2 that has a completely different structure compared with reported ones, and provide a molecular framework, Bcl-2-caspase 3-HDAC6, to understand the complexity of mutual regulation between apoptosis and autophagy.

## 2. Results

### 2.1. Spiroindoline Compound ***6d***, as an Effective Anticancer Inhibitor, Is Capable of Inhibiting CRC Cell Proliferation and Growth In Vitro Cell Model and In Vivo Xenograft Model

**Figure 1:**
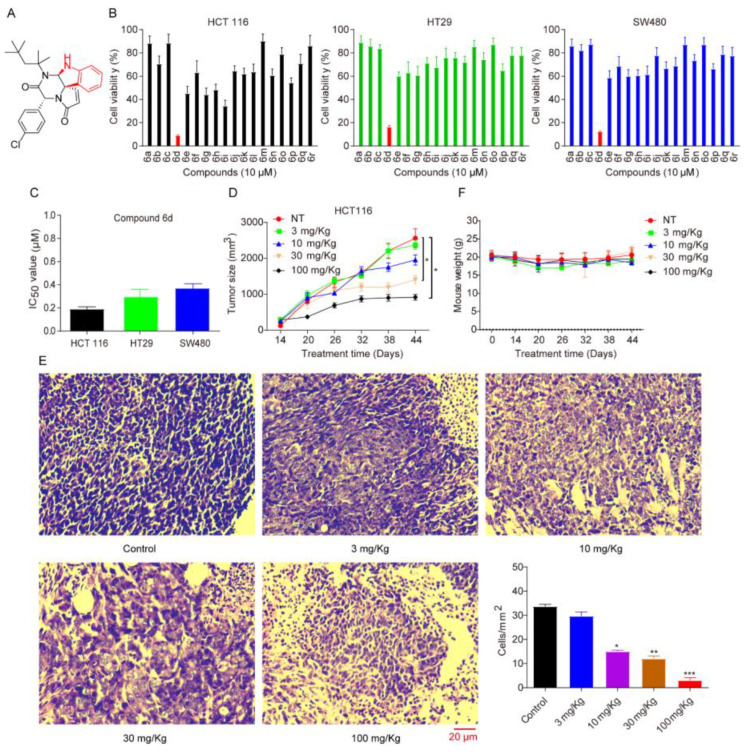
Spiroindoline compound **6d** decreases the viability of CRC cell lines. (**A**) The chemical structure of compound **6d**. (**B**) The bioactivity of compound **6d** was assessed by MTT in CRC cells, including HCT116, HT29 and SW480. These cells were treated with compound **6d** at 10 μM for 48 h, respectively, and then cell viability was measured. Data were the mean ± SD of three independent experiments, and each experiment was conducted in sextuplicate. (**C**) The IC_50_ value of compound **6d** was measured and calculated in CRC cell lines. (**D**) SCID/Nude mice bearing HCT116 tumor xenografts were administrated with or without compound **6d** by intraperitoneal manner. Mean tumour volume or weight ± SD was shown; n = 6 mice per group. (**E**) Representative images of hematoxylin and eosin (H&E) staining in HCT116-induced tumor xenografts. Scale bar indicates 20 μm. (**F**) The body weight of the mice from each group was not remarkably influenced by the compound **6d** treatment, suggesting that there was no significant toxicity. All data are presented as the mean ± SD of three independent experiments. * *p* < 0.05; ** *p* < 0.01; and *** *p* < 0.001 versus vehicle.

Previously, we have demonstrated that compound **6d** can significantly reduce the cell proliferation efficiency of a panel of human tumor cell lines, including glioma cell U87, pancreatic cancer cell PANC-1, prostate cancer cell PC3, hepatoma cell Hep3B, lung adenocarcinoma cell A549, and breast cancer cell MCF7 [27]. The chemical structure of compound **6d** is shown in Figure 1A. To further explore whether compound **6d** exerts a similar effect on CRC, we performed the MTT assay and found that compound **6d** can significantly decrease the CRC cell viability at 10 μM compared to compound **6a**–**6c**, **6e**–**6r** (Figure 1B). In contrast, only mild cytotoxicity was associated with exposure to compound **6d** in the normal adult colonic epithelial cell line, FHC (Figure S1). The IC_50_ values of compound **6d** in HCT116, HT29, SW480, and FHC cells were 0.21, 0.33, 0.42, and 2.8 μM, respectively (Figure 1C and Figure S1). Furthermore, to assess the antitumor effect of compound **6d**, SCID/Nude mice bearing subcutaneous HCT116 tumor cell xenografts were established, the tumor growth rate was monitored, and then compound **6d** was administrated into mice with intraperitoneal injection every 3-day intervals for 36 days when tumor volume reached around 100 mm^3^. Notably, compound **6d** treatment at concentrations of 30 and 100 mg/kg substantially suppressed the tumor growth of human CRC after 36 days in a dose-dependent manner compared with vehicle-treated control mice (Figure 1D). Consistently, hematoxylin and eosin (H&E) staining data indicated a significant decrease in cell density after treatment with compound **6d** (Figure 1E). Importantly, no systemic toxicity was detected during treatment because no mortality or loss of body weight was observed for each group of model mice as shown in Figure 1F. Together, these results showed that compound **6d** could suppress cell proliferation and tumorigenicity in vivo, implying that compound **6d** has the potential as an antitumor inhibitor for the treatment of human CRC.

### 2.2. Compound ***6d*** Induces the Mitochondrial-Dependent Apoptotic Pathway through Pharmacolog Ical Inhibiting Bcl-2

**Figure 2:**
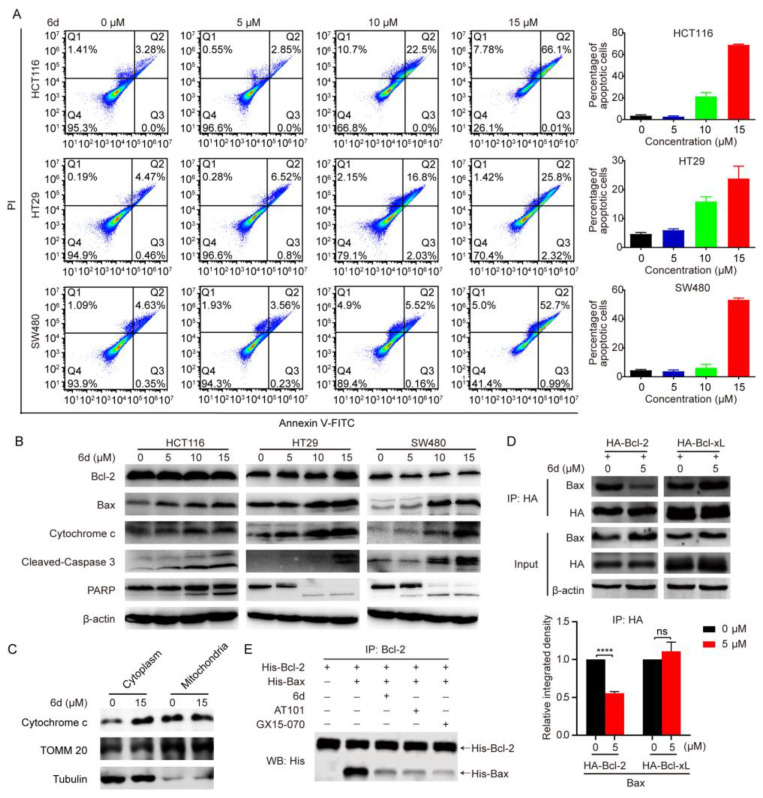
Compound **6d** induces mitochondrial-mediated intrinsic apoptosis by targeting Bcl-2. (**A**) CRC cells were treated with indicated concentrations of compound **6d** for 8 h and then stained with the Annexin V-FITC/PI kit. The stained cells were analyzed with flow cytometry analysis. (**B**) Compound **6d** regulates the expression of apoptosis-related proteins. CRC cells were treated with different concentrations of compound **6d** (0, 5, 10, 15 μmol/L) for 8 h. The release of cytosol cytochrome c, expression of the BCL2 family proteins Bcl-2 and Bax, the cleavage levels of activated caspase 3 and poly (ADP-ribose) polymerase (PARP), were estimated to determine whether or not the intrinsic apoptotic pathway is involved in the anti-tumor effect. β-actin level was used as internal control for equal protein loading. (**C**) Compound **6d** treatment increased cytosolic cytochrome c levels. Control and compound **6d** (15 μM)-treated cells were subjected to cell fractionation using kit to separate cytoplasmic and mitochondrial fraction, and then cytosolic and mitochondrial cytochrome c levels were detected through western blotting, respectively. The translocase of outer mitochondrial membrane 20 homolog (Tomm 20) was used as a mitochondrial marker. (**D**) Compound **6d** inhibits the interaction between exogenous Bcl-2 and endogenous Bax, but not the interaction of Bcl-xL and Bax. After treatment, lysates from HCT116 cells transfected with HA-Bcl-2 or HA-Bcl-xL were pulled down with anti-HA, followed by WB with corresponding primary antibodies. **** *p* < 0.0001 versus vehicle. (**E**) In vitro binding assay was performed using recombinant His-Bcl-2 and His-Bax proteins. Anti-Bcl-2 antibody was used for pull-down after incubating recombinant proteins with compound **6d**, AT101 and GX15-070, and then anti-His antibody was employed for WB. Both AT101 and GX15-070 were used as positive control.

To gain more insight into the mode of anti-tumor effect of compound **6d** on CRC cells, we determined whether or not the intrinsic pathway of apoptosis is involved in the process. An Annexin V-FITC/PI assay was employed via flow cytometry after CRC cells exposure to compound **6d** for 8 h, and typical images and histograms in Figure 2A showed that compound **6d** significantly induced cellular apoptosis in all CRC cells and the proportion of late-phase apoptosis was dramatically increased in a dose-dependent manner (from 2.85% to 66.1% for HCT 116 cells; from 4.47% to 25.8% for LN229 cells; from 4.63% to 52.7% for SW480 cells). Furthermore, we evaluated the effect of compound **6d** on the expression of Bcl-2 family members and cytochrome c to describe the mechanism underlying compound **6d**-induced apoptosis. As shown in Figure 2B and Figure S2, cells treated with compound **6d** led to an increase in Bax and cytochrome c, while Bcl-2 remained substantially unchanged. The release level of cytochrome c from mitochondria to cytoplasm is the gold standard to reflect the mitochondria-mediated apoptotic level. Therefore, we isolated the cytosolic and mitochondrial fractions, respectively, to evaluate the levels of cytochrome c. The result showed that compound **6d** treatment increased cytosolic cytochrome c levels, which implied that compound **6d** treatment can increase cytochrome c releasing from mitochondria to cytoplasm (Figure 2C). Next, to confirm the induction of mitochondrial-related apoptosis caused by compound-**6d**, we evaluated the levels of activated caspase 3 and PARP, and found that the cleaved forms of caspase 3 and PARP were notably increased in CRC and other cancer cells (such as A549, U87, and PANC-1) after treatment with compound **6d**, but not in MCF7 cells due to a deletion mutation in exon 3 of the caspase 3 gene [28] (Figure 2B and Figure S2).

Given that Bax could directly bind to Bcl-2 by its BH3 domain, and our above data related to cytochrome c release from mitochondria and caspase activation-mediated apoptosis, we hypothesized that compound **6d** may play an antitumor effect by targeting Bcl-2. If so, the binding between Bcl-2 and Bax would decrease after exposure to compound **6d**. HCT116 cells were transfected with exogenous Flag-Bcl-2 and detected the localization of Flag-Bcl-2 and Bax using immunofluorescent assay. The result showed that Flag-Bcl-2 co-localized well with Bax, which localizes at the mitochondrial membrane (Figure S3). As expected, the degree of interaction between exogenous HA-Bcl-2 and endogenous Bax was markedly attenuated in the compound **6d** treatment group compared to the DMSO control (Figure 2D). To further substantiate the Bcl-2-Bax binding inhibition results of compound **6d**, we carried out an in vitro pull-down assay using the highly purified recombinant protein His-Bcl-2 and His-Bax, and obatoclax (GX15-070) and (R)-(-)-Gossypol (AT101), two small-molecule BH-3 mimetics that antagonize Bcl-2, as positive control. To support this hypothesis, similar to the effect exerted by GX15-070 and AT101, exposure to compound **6d** resulted in a remarkable reduction in the binding of Bcl-2 and Bax (Figure 2E), suggesting that compound **6d** may directly bind to Bcl-2 and act as a novel potential inhibitor of Bcl-2. Collectively, these results indicate that compound **6d** may act as a novel potential inhibitor to dissociate Bcl-2 from Bax, and then promotes the mitochondrial-dependent apoptotic pathway.

### 2.3. Compound ***6d*** Initiates Autophagosome Formation by Disrupting the Interaction between Bcl-2 and Beclin 1

**Figure 3:**
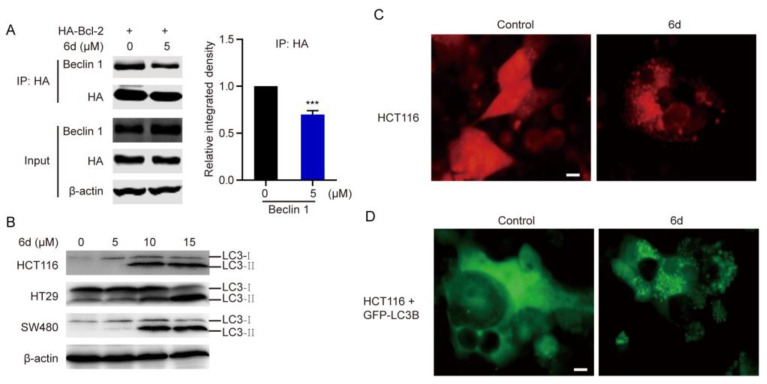
Compound **6d** initiates autophagy and contributes to the formation of autophagosomes by releasing Beclin 1 from Bcl-2. (**A**) HCT116 cells were transfected with HA-Bcl-2, and then treated with or without 5 μM compound **6d** for 8 h. Co-IP was performed using HA antibody to detect the interaction between Bcl-2 and Beclin-1. *** *p* < 0.001 versus vehicle. (**B**) Western blotting analysis of LC3B in CRC cells treated with indicated concentrations of compound **6d** for 8 h. β-actin level was used as an internal load control. (**C**) Immunofluorescence analysis of the endogenous LC3 dots signal in HCT16 cells treated with or without compound **6d** for 4 h and stained with LC3B antibody. Red signal indicated the endogenous LC3B puncta formation. (**D**) Fluorescence analysis of exogenous LC3 dots signal in HCT16 cells transfected with GFP-LC3 after treatment with or without compound **6d**. Scale bar: 10 μm.

A vital event in the autophagy process is the formation of isolated membranes, which is regulated by the activation of the Beclin1-Vps34 complex. Bcl-2 functions as both an anti-apoptotic and anti-autophagic regulator, and interacts with Beclin1 to inhibit Beclin1-dependent autophagy [19]. Considering that compound **6d** may be an inhibitor targeting Bcl-2, we further explored whether the interaction between Bcl-2 and Beclin 1 was influenced after treatment with compound **6d**. As indicated in Figure 3A, the interaction between Beclin-1 and Bcl-2 was affected in response to treatment with compound **6d**. The blockage of interaction between them suggests the possibility that compound **6d** might be an initiator of autophagy. Next, we detected autophagy activation by monitoring the conversion from the cytosolic form, LB3B-I, to the autophagosome-associated form, LB3B-II, a specific and conserved marker of autophagy. As hypothesized, we found that conversion to LB3B-II is significantly induced in CRC cells and other cancer cells (including MCF7, U87, Hep3B, and PANC-1) upon compound **6d** treatment (Figure 3B and Figure S4), indicating that autophagy was activated and autophagosomes were formed after treatment. Furthermore, endogenous LC3 puncta, representing autophagic vacuoles, were considerably increased in compound **6d** treated CRC cells (Figure 3C). Consistently, the number of exogenous GFP-LC3B puncta was remarkably increased following treatment, suggesting an ability to induce autophagosome accumulation (Figure 3D). In summary, these results suggest that compound **6d** may compete with Beclin 1 to bind to Bcl-2, thereby inducing autophagy and the formation of autophagosomes.

### 2.4. Compound ***6d*** Blocks Autophagic Flux by Promoting Histone Deacetylase-6 (HDAC6) Cleavage

**Figure 4:**
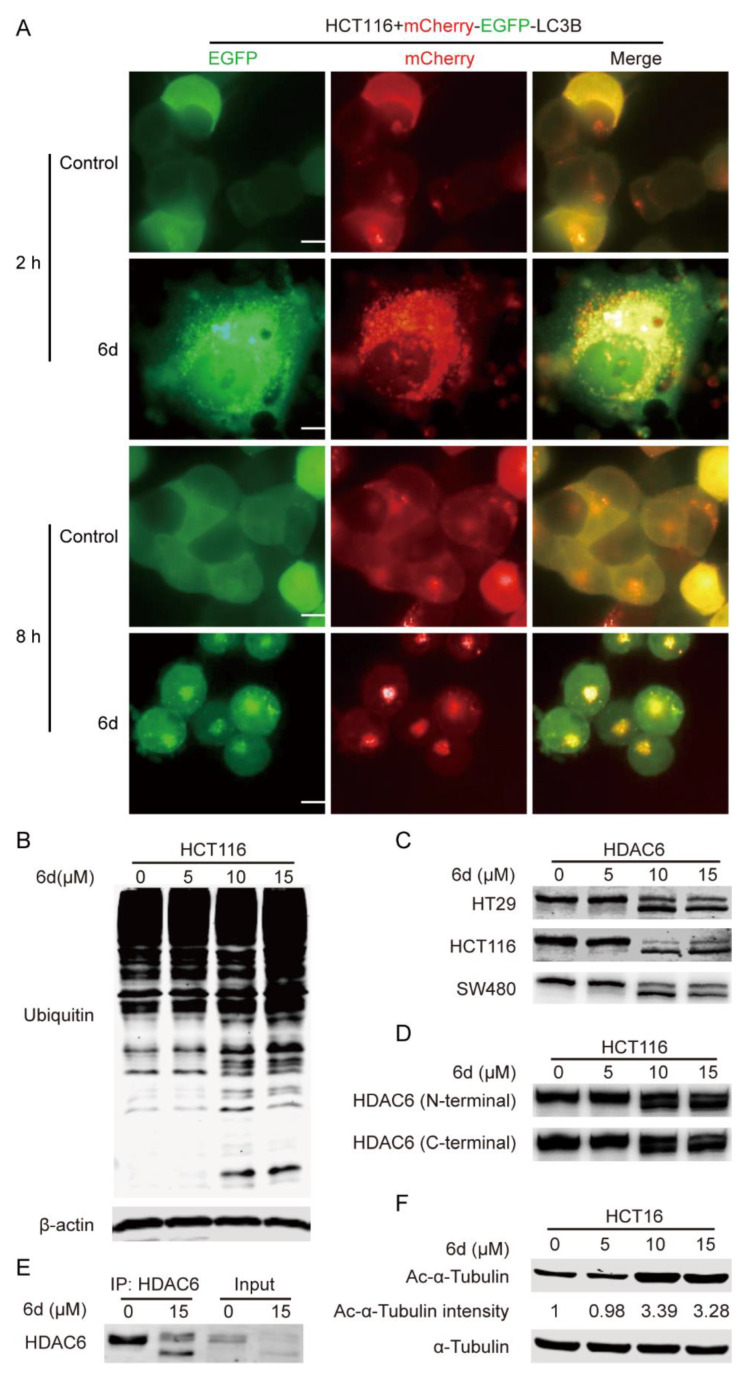
HDAC6 cleavage induced by compound **6d** influences its deacetylase activity, autophagic flux and ubiquitinated protein degradation. (**A**) The tandem mCherry-GFP tagged LC3B pH-based autophagy reporter system was used to analyze the effect of compound **6d** on autophagic flux. Autophagosomes appear as yellow puncta (both mCherry and GFP signals), while autophagolysosomes appear as red puncta (mCherry-only signals). Fluorescent images of mCherry-GFP-LC3B puncta in HCT116 cells treated with compound **6d** were captured by an opera^®^ high-throughput confocal imaging platform. Scale bar, 10 μm. (**B**) Compound **6d** promotes the accumulation of ubiquitinated proteins in a dose-dependent manner. β-actin level was used as internal control for equal protein loading. (**C**) Compound **6d** induces the cleavage of HDAC6 in CRC cells. Lysates were harvested from HT29, HCT116, and SW480 cells after exposure to the indicated concentrations of compound **6d**, and then subjected to WB with antibody against the N-terminal (1–100 aa) of HDAC6. (**D**) Both the antibodies against N- or C-terminal of HDAC6 are capable of recognizing the cleaved fragments induced by compound **6d**. (**E**) IP with antibody against the N-terminal of HDAC6 confirms the HDAC6 cleavage triggered by compound **6d** in HCT116 cells. (**F**) The acetylation of α-tubulin was significantly elevated in a dose-dependent manner in HCT116 cells treated with compound **6d**.

**Figure 5:**
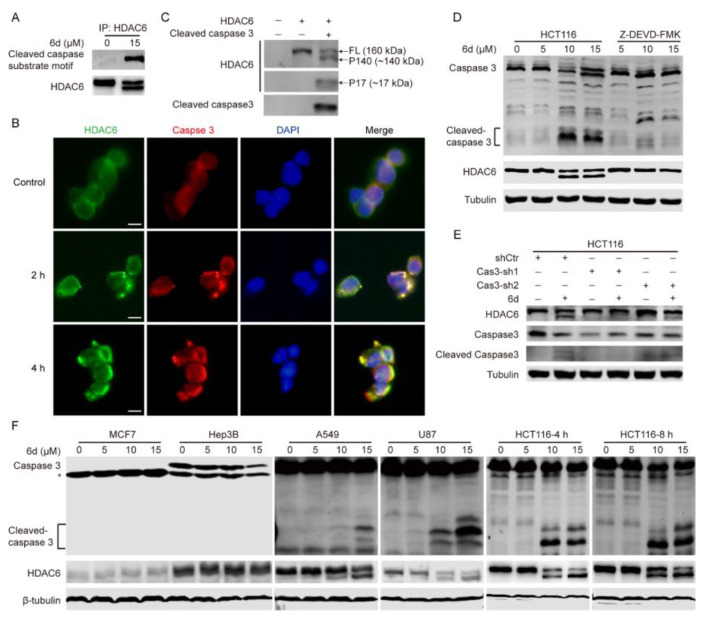
HDAC6 is a direct substrate of caspase 3 and is cleaved by caspase 3 in response to compound **6d**. (**A**) HDAC6 is cleaved by caspases in the presence of compound **6d**. A cleaved caspase substrate motif (DE (T/S/A)D) antibody was used to identify endogenous levels of caspase-cleaved proteins with a C-terminal aspartic acid residue. IP was performed using an anti-HDAC6 antibody, and was then subjected to WB with the cleaved caspase substrate motif (DE (T/S/A)D) antibody. (**B**) Compound **6d** promotes the punctuated accumulation of caspase 3 and co-localization between caspase 3 and HDAC6. After 12 h of incubation with compound **6d**, HCT116 cells were fixed, permeabilized, and stained with anti-HDAC6 antibody (green), anti-caspase 3 antibody (red), and DAPI (blue). Scale bar, 10 μm. (**C**) HDAC6 is a direct substrate of caspase 6. In vitro cleavage experiment was employed using exogenous expressed HDAC6 and cleaved caspase 3, the active form of caspase 3. The samples were analyzed using WB with corresponding antibodies after 2 h of incubation. FL: full-length of HDAC6; P140: a ~140 kDa long-cleaved band of HDAC6; P17: a ~17 kDa short-cleaved band of HDAC6. (**D**) Full-length of HDAC6 was recovered in the presence of caspase 3 inhibitor. HCT116 cells were treated with compound **6d** for 4 h, and these cells were subsequently exposed in the absence and presence of the caspase 3 inhibitor Z-DEVD-FMK (20 μM). Following that, the lysates were collected and subjected to WB with indicated antibodies. β-tubulin was loaded as a control. (**E**) Caspase 3 depletion eliminates the effect of compound **6d** on HDAC6 cleavage. HCT116 cells were transfected with caspase 3 shRNA and scramble shRNA, and were then subjected to WB after treatment with or without compound **6d** for 4 h. (**F**) HDAC6 cleavage induced by compound **6d** depends on the presence and activation of caspase 3. Different cancer cells, such as MCF7 (absence of caspase 3), Hep3B, A549, U87, and HCT116, were treated with indicated concentrations of compound **6d**, and then subjected to WB using the corresponding antibodies. “*” represented non-specific band.

Autophagy, a major intracellular degradation system, can deliver cytoplasmic cytotoxic misfolded protein aggregates to and degrade in the lysosome [29]. Impaired autophagy machinery may be a disaster to cancer cells and result in apoptosis and cell death due to the accumulation of misfolded proteins and damaged organelles [30]. To further evaluate the effect of compound **6d** on the autophagic flux process, a PH-sensitive double tagged mCherry-GFP-LC3B reporter was used to examine the fusion efficiency. The yellow fluorescence represented the number of non-acidic autophagosomes, while the red fluorescence represented labeled autophagolysosomes. As shown in Figure 4A, a significant increase in the number of yellow, fluorescent vesicles in compound **6d** treated HCT116 cells as compared with the control, indicating the accumulation of autophagosomes caused by a defect of fusion between autophagosome and lysosome. Furthermore, we also found that compound **6d**-treated cells accumulated ubiquitinated proteins at a higher level than those without treatment (Figure 4B), implying that blockage of autophagic flux results in the failure of aggregates degradation. It was reported that HDAC6 promotes the fusion of autophagosome and lysosome to control ubiquitin-selective quality-control autophagy [25]. To explore whether the underlying mechanism involved in the inhibition of autophagosome and lysosome fusion caused by compound **6d** is associated with HDAC6, the expression level and activity of HDAC6 were detected using western blotting in different cancer cells. Interestingly, double-HDAC6 bands were observed in CRC, U87, A549, and PANC-1 cells treated with compound **6d** as compared to the control, while the cleaved band was not observed in Hep3B and MCF7 cells regardless of treatment or nontreatment (Figure 4C and Figure 5F). Additionally, to further confirm these observations, both N (amino)-terminal (specifically against the fragment within HDAC6 1–100 amino acids (aa)) and C (carboxyl)-terminal antibodies of HDAC6 were used to detect HDAC6. Consistent with the above data, both N- and C-terminal antibodies can recognize double bands after compound **6d** treatment (Figure 4D and Figure S5A), indicating the truthful proteolytic cleavage of HDAC6 induced by the inhibitor at C-terminal, but not at N-terminal. Immunoprecipitation (IP) was also used to evaluate the cleaved band of HDAC6, and the result showed that the proteolytic cleavage of HDAC6 presented in the compound **6d**-treated HCT116 cells (Figure 4E). Notably, qRT-PCR (Quantitative reverse transcription-polymerase chain reaction) analysis revealed that the mRNA level of HDAC6 was not influenced after treatment with compound **6d** (Figure S5B), suggesting that the cleavage of HDAC6 occurs at a protein level rather than at a transcriptional level. Next, we further evaluated the effect of compound **6d** on the deacetylase activity of HDAC6, and found that the acetylation of α-tubulin, a faithful substrate of HDAC6, increased significantly in a dose-dependent manner in HCT116 cells (Figure 4F). To gain further evidence as to whether compound **6d** can cleave other HDACs, we analyzed the cleavage of HDAC1, HDAC2, HDAC3, HDAC4, HDAC5, and HDAC7 induced by compound **6d**. As shown in Figure S6, compound **6d** specifically induced cleavage of HDAC6 into double bands compared to other HDACs, confirming its more selective effect on HDAC6 cleavage. These data suggest that the blockage of compound **6d**-trigged autophagic flux and the accumulation of ubiquitinated proteins were mediated by HDAC6 cleavage and its inactivation.

### 2.5. HDAC6 Serves as a Substrate of Caspase 3 and Compound ***6d*** Specifically Induces HDAC6 Cleavage by Activating Caspase 3

**Figure 6:**
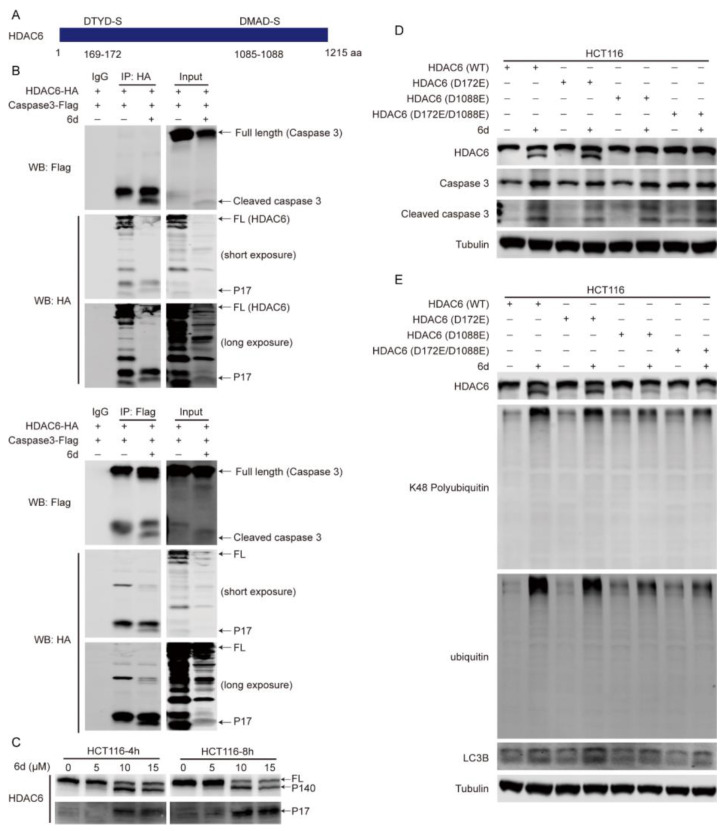
HDAC6 is truncated by caspase 3 at D1088 site. (**A**) Diagram showing the putative caspase 3 cleavage motifs in HDAC6. (**B**) HDAC6 is cleaved by caspase 3 at C-terminal. HCT116 cells were transfected with HDAC6-HA (HA-tagged at the C-terminal of HDAC6) and caspase 3-Flag (Flag-tagged at the C-terminal of caspase 3), and treated with or without compound **6d**, followed by HA or Flag for IP and corresponding primary antibodies for WB. (**C**) Endogenous HDAC6 was truncated into ~140 kDa and ~17 kDa fragments. HCT116 cells were exposed to the indicated concentrations of compound **6d** for 4 h and 8 h, respectively, and then the lysates were subjected to WB. (**D**) HDAC6 was cleaved at D1088 within the C-terminal in the presence of compound **6d**. HCT116 cells transfected with wild-type (WT) or mutated HDAC6 plasmids were treated with compound **6d**, and then HDAC6, caspase 3, cleaved caspase 3 and tubulin were detected by WB in total cell lysates. (**E**) The mutation at D1088 site of HDAC6 weakens the effect of compound **6d** on the accumulation of K48-linked ubiquitinated proteins and LC3B. The transfected HCT116 cells were exposed to compound **6d**, and followed by WB using anti-ubiquitin, specific anti-K48-linked ubiquitin, and anti-LC3B antibodies.

Next, we aimed to explore the potential molecular mechanism underlying the compound **6d**-induced HDAC6 cleavage. Having detected the noticeable activation of caspase 3 induced by compound **6d**, we assessed whether caspase 3 was involved in the cleavage of HDAC6. A cleaved caspase substrate motif (DE (T/S/A)D) antibody was used to identify endogenous levels of caspase-cleaved proteins with a C-terminal aspartic acid residue. As shown in Figure 5A, this antibody only recognized the ~140 kDa cleaved-band (referred to hereafter as P140) in the sample treated with 15 μM compound **6d** rather than the full length (FL) of HDAC6 in both treated and untreated HCT116 cells. Furthermore, co-immunoprecipitation (Co-IP) showed that caspase 3 interacted with HDAC6 regardless of the presence or absence of compound **6d** as compared with control, and the interaction between them was slightly elevated after compound **6d** treatment (Figure 6B). Consistently, the indirect immunoflurescence assay indicated that there was less co-localization between HDAC6 (green signal) and caspase 3 (red signal) in the control cells, while significant co-localization of them was observed in cells treated with compound **6d** for 2 h and 4 h (Figure 5B). To obtain direct evidence that caspase 3 is the enzyme for cleaving HDAC6, an in vitro cleavage assay was conducted using exogenous proteins HDAC6 and cleaved caspase 3. Of note, in comparison with control, the activating form of caspase 3, cleaved caspase 3, can remarkably cleave the full-length of HDAC6 into two fragments, a ~140 kDa long-cleaved band (P140) and a ~17 kDa short-cleaved band (referred to hereafter as P17), respectively (Figure 5C). Using a caspase 3 inhibitor, Z-DEVD-FMK, we demonstrated that the full-length of HDAC6 was completely restored compared with compound **6d** alone in HCT116 cells during concurrent treatment with compound **6d** and Z-DEVD-FMK (Figure 5D). Consistently, we assessed the cleavage of HDAC6 in caspase 3 knockdown and control cells in response to compound **6d** and found that caspase 3 attenuation by siRNA completely prevented cleavage of HDAC6 in the treatment of compound **6d** (Figure 5E). Importantly, we found that different cancer cell lines, such as A549, U87, Hep3B, PANC-1, and HCT116, expressed high level of caspase 3 but not MCF7, and the cleaved caspase 3 was notably enhanced in A549, U87, PANC-1, and HCT 116 cells after treatment with compound **6d**, while it was not cleaved in Hep3B cells (Figure 5F). As expected, double-HDAC6 bands were only observed in U87, A549, and HCT116 cells treated with compound **6d** as compared to untreated cells, whereas there were no cleaved band in Hep3B and MCF7 cells regardless of treatment or nontreatment (Figure 5F), indicating that compound **6d**-induced HDAC6 cleavage is dependent on caspase 3 activation. In summary, these results strongly suggest that caspase 3 is a veritable enzyme for HDAC6 cleavage and compound **6d** specifically induces prior caspase 3 activation by targeting Bcl-2, thereby promoting HDAC6 cleavage.

### 2.6. The D1088 Localized in the DMAD-S Motif at C-Terminus of HDCA6 Is the Actual Site for Proteolytic Cleavage by Caspase 3

Since HDAC6 cleavage by caspase 3 was detected in the presence of compound **6d**, we inferred that there are some potential caspase 3 cleavage sites within HDAC6. As expected, we analyzed the HDAC6 sequence for the presence of putative cleavage motifs through the bioinformatics website (https://www.dmbr.ugent.be/prx/bioit2-public/SitePrediction (accessed on 24 September 2021)) and identified two main potential caspase 3 cleaved motifs (DXXD), DTYD-S and DMAD-S, which are located at the N-terminus (169–172 aa) and C-terminus (1085–1088 aa), respectively (Figure 6A). To validate the prediction, we therefore used transfection with caspase 3-Flag and HDAC6-HA (HA-tagged at C-terminus of HDAC6) and followed Co-IP pull-down with anti-HA/anti-Flag antibodies and WB detection probed with anti-Flag/anti-HA antibodies to evaluate HDAC6 cleavage by caspase 3. We found that only HDAC6 FL and P17 were detected using anti-HA antibody regardless of anti-HA or anti-Flag IP but we did not detect P140 (Figure 6B). In line with this, the endogenous P17 and P140 species of HDAC6 were highly enriched in compound **6d**-treating cells compared to control, whereas its FL form was obviously reduced (Figure 6C).Thus, P17 appeared to be a small C-terminal fragment of HDAC6, whereas P140 may be an N-terminal one, indicating that compound **6d**-induced cleavage occurs within the C-terminal rather than N-terminal of HDAC6.

To test whether HDAC6 is cleaved at the DTYD-S or DMAD-S site by caspase 3, we mutated D172 and D1088 to glutamic acid (D172E and D1088E) alone or together with vectors containing full-length human HDAC6. Full-length HDAC6 (Wild-type, WT), D172E, D1088E, and D172E/D1088E vectors were transiently transfected into HCT116 cells, and WB was performed with anti-HDAC6 or anti-caspase 3 antibodies after exposure to compound **6d**. The HDAC6 FL protein and the low level of P140 products appeared in the WT and D172E groups, whereas D1088E and D172E/D1088E eliminated the existence of truncated P140 species (Figure 6D). Similarly, caspase 3 was remarkably activated in all transfected groups treated with compound **6d**, implying that HDAC6 is cleaved at D1088 by caspase 3. It has been reported that HDAC6 associates with and clears ubiquitinated protein aggregates through its uncommon ubiquitin-binding domain, the zinc-finger ubiquitin binding domain (BUZ) [23,25]. To explore whether HDAC6 cleavage induced by compound **6d** can affect the accumulation of ubiquitinated proteins, HCT116 cells were transfected with WT, D172E, D1088E, or D172E/D1088E vectors of HDAC6 followed by WB using anti-ubiquitin or anti-K48-specific ubiquitin antibody, respectively. Interestingly, the expression of wild-type HDAC6 and D172E led to a notable accumulation of ubiquitinated proteins accompanied by obvious HDAC6 cleavage and LC3B accumulation after compound **6d** treatment, especially the K48-linked ubiquitinated proteins. In contrast, the mutations at D1088E and D172E/D1088E promote weak accumulation of total ubiquitinated proteins, K48-linked ubiquitinated proteins, or LC3B after exposure to this compound (Figure 6E), indicating that HDAC6 cleavage at D1088 loses the ability to associate and clear ubiquitinated protein aggregates. Taken together, these results suggest that HDAC6 is specifically cleaved at D1088 by caspase 3, which is activated by compound **6d**, and then leads to the accumulation of ubiquitinated protein aggregates.

## 3. Discussion

Although there were several preclinical and clinical studies related to small molecular inhibitors against Bcl-2, targeting Bcl-2 could result in the development of resistance to Bcl-2 inhibitors [15,31,32]. Many of the reported Bcl-2 inhibitors selectively targeted its well-conserved BH3 domain [33]. Site mutations, such as G101V, D103Y, and F104I, influenced the BH3-binding pocket of Bcl-2 protein, and subsequently led to the off-target effect [34,35,36]. Obviously, the specificity of Bcl-2 inhibitor, venetoclax, was conferred by a hydrogen bond involving D103 in the BH3 domain of Bcl-2, whereas D103 was displaced by E103 in Bcl-xL [33]. Therefore, binding an inhibitor to different residues in the hydrophobic groove can be an effective strategy not only for overcoming therapeutic resistance but also for solving BCL2 specificity. In this study, we reported the identification and characterization of a novel inhibitor targeting Bcl-2. The chemical structure of the identified inhibitor of Bcl-2 was completely different from the reported ones. However, our competitive binding results showed that compound **6d** significantly suppressed the interaction between Bcl-2 and Bax by binding to Bcl-2, and then triggered mitochondria-mediated intrinsic apoptosis to inhibit the proliferation and growth of CRC cells in vitro/vivo, suggesting that the effects of compound **6d** are similar with those exhibited by reported inhibitors of Bcl-2. Although we do not know the binding sites for the binding of compound **6d** to Bcl-2, these findings imply that its binding sites with Bcl-2 may be different from the site locations of other inhibitors due to completely different chemical structures of them.

Apoptosis and autophagy were thought to be two mutually cross-regulated cellular events as they share several critical molecular regulators, such as JNK1, Bcl-2, and Beclin 1 [37,38]. The anti-apoptotic protein Bcl-2 interacted with the BH3 domain of Beclin 1 to impede the formation of Beclin 1–hVps34–hVps15 core complex, and then attenuated the Beclin 1-dependent autophagy-activated pathway [39]. Caspases are proteolytic enzymes and function as molecular switches between autophagy and apoptosis by cleaving several autophagy-related proteins. It has been reported that Beclin 1 can be cleaved by various caspases, such as caspase 3, 6, 8, and 9, and truncated into multiple fragments with different sizes of molecular weights [40,41,42]. As a result, Beclin 1-dependent autophagy initiation was suppressed [43]. Additionally, caspase-mediated apoptosis has been proposed to reduce autophagy by recognizing and cleaving ATGs (Autophagy-related proteins) [44]. Conversely, the deficiency of autophagic flux could trigger cell apoptosis resulting from the accumulation of cytotoxic misfolded protein aggregates, further complicating the interpretation of results that involve both autophagy and apoptosis [22,23]. However, there has been little research related to the involvement of Bcl2 and caspases in regulating fusion between autophagosome and lysosome. In our study, we found that HDAC6 is a faithful substrate of caspase 3, and pharmacological targeting Bcl-2 by compound **6d** activates caspase 3 followed by HDAC6 cleavage. As is reported in previous studies, HDAC6 promotes the transport of ubiquitinated protein aggregates to generate aggresomes through the microtubule network [23] and improves their subsequent clearance by triggering the fusion between autophagosomes and lysosomes through its unique association with the actin cytoskeleton [25]. Consistent with previous reports, our results indicated that ubiquitinated proteins and autophagosomes are accumulated after HDAC6 cleavage mediated by caspase 3 activation. Our findings innovatively indicated that apoptosis-related proteins Bcl-2 and caspase 3 are involved not only in autophagy initiation but also in regulation of the ubiquitinated protein degradation and the fusion of autophagosomes and lysosomes.

The sequence structure of HDAC6 is composed of a nuclear localization signal (NLS), a nuclear export signal (NES), two tandem deacetylase domains (DD1 and DD2), a dynein-binding domain (DMB) located in the linker region between DD1 and DD2, which directly binds to motor protein dynein, a cytoplasmic retention domain (SE14), and a BUZ domain that locates at the C-terminus from the 1131 aa to end and binds ubiquitinated misfolded proteins via the C-terminus glycine-glycine residues of ubiquitin [45]. The caspase-3 cleavage site identified in our study is present between the SE14 and BUZ domains, and the cleavage eliminates the entire BUZ domain from HDAC6. This finding is consistent with a previous report that indicated that HDAC6 was a substrate of caspase 3 and the cleavage site of HDAC6 by caspase 3 localized at D1088 [46]. To our surprise, the deacetylase activity of HDA6 and its interaction with microtubule and actin are significantly reduced after exposure to compound **6d** demonstrated by up-regulated acetylation of substrate α-tubulin and suppression of fusion between autophagosomes and lysosomes. As of yet, we do not know the real mechanism underlying how the removal of the BUZ domain from HDAC6 influences its biological functions. The possible reasons for this are: (1) HDAC6 losing the BUZ domain leads to its conformation variation, and then inactivates deacetylase activity and blocks the autophagic flux process; (2) HDAC6 combined with activated caspase 3 may affect the binding affinity of HDAC6 with its substrates for deacetylation or microtubule and actin cytoskeleton for the fusion of autophagosomes and lysosomes; (3) After treatment with compound **6d**, there may be some undiscovered molecules which are capable of influencing enzyme activity of HDAC6 and interaction between HDAC6 and microtubule and actin cytoskeleton. However, the detailed mechanisms need to be further investigated and verified.

In conclusion, our findings presented in this study uncover a crucial interplay among anti-apoptotic protein Bcl-2, apoptotic executor caspase 3 and deacetylase HDAC6, and identify HDAC6 as a direct substrate for caspase 3. Of note, this study provides a novel Bcl-2 inhibitor with completely different structures compared to the reported ones. Compound **6d** can induce caspase 3-mediated mitochondrial intrinsic apoptosis by targeting Bcl-2 to exert its antitumor viability in vivo/vitro, and then caspase 3 activation directly cleaves HDAC6 into fragments with 140 kDa and 17 kDa. Moreover, the cleavage site is located at D1088 in the DMAD-S motif at C-terminus of HDAC6. Interestingly, compound **6d** not only initiates autophagosome formation by inhibiting the interaction between Bcl-2 and Beclin 1 but also hinders fusion between autophagosomes and lysosomes via caspase 3-dependent HDAC6 cleavage. Our findings favorably implied the anti-tumor mechanism of compound **6d** and offered evidence of its potential as a promising new lead compound in the discovery of chemotherapeutic agents.

## 4. Materials and Methods

### 4.1. Antibodies, Plasmids, and Reagents

Antibodies against Caspase 3 (9662S), Cleaved Caspase 3 (9664S), Bcl2 (15071T), Bax (41162S), Cytochrome c (4280S), Acetyl-α-Tubulin (Lys40) (5335S), α-Tubulin (3873S), β-Tubulin (2146S), LC3B (83506S), Beclin (3495S), K48-linkage Specific Polyubiquitin (8081S), Ubiquitin (3936S), HA-Tag (3724S), PARP (9532S), β-Actin (3700S), Cleaved Caspase Substrate Motif [DE(T/S/A)D] (8698S), HDAC6 (7558S), Class I HDAC Antibody Sampler Kit (65816T), Flag (14794S), and Class II HDAC Antibody Sampler Kit (7989T) were purchased from Cell Signaling Technologies (Danvers, Massachusetts, USA). HDAC6 (N-terminal, 1–100 aa) (ab133493) antibody was obtained from Abcam (Cambridge, UK). Anti-Tomm 20 (11802-1-AP) was purchased from Proteintech (Chicago, IL, USA). The plasmid constructs expressing pCDH-CMV-Bcl-2-HA-coGFP-Puro, pCDH-CMV-Bcl-xL-HA-coGFP-Puro, pCDH-CMV-HA-HDAC6-3×Flag-coGFP-Puro, pCDH-CMV-HA-HDAC6 (D1088E)-3×Flag-coGFP-Puro, pCDH-CMV-HA-HDAC6 (D172E)-3×Flag-coGFP-Puro, pCDH-CMV-HA-HDAC6 (D172E/D1088E)-3×Flag-coGFP-Puro, pCDH-CMV-Caspase 3-3×Flag-coGFP-Puro, Lenti-mCherry-GFP-LC3B (Lentivirus expressing mCherry-EGFP-LC3B fusion protein), and the lentiviral Caspase 3-shRNA vectors, and their control vectors were custom-made from VigeneBio, Shandong Branch (WZ Biosciences Inc., Jinan, China). pEnCMV-Bcl-2-Flag-SV40-Neo (P24963) and pCDH-CMV-EGFP-Linker-LC3B-EF1a-Puro (P23666) were obtained from MiaolingPlasmid (Wuhan, China). Compounds such as Z-DEVD-FMK (S7312), Obatoclax (GX15-070) (S6709), and (R)-(-)-Gossypol acetic acid (AT101) (S2812) were purchased from Selleckchem (Houston, TX, USA).

### 4.2. Cell Lines and Culture

HEK293T, A549, MCF7, U87, HCT116, HT29, SW480, PANC-1, and Hep3B were acquired from American Type Culture Collection (ATCC, Manassas, VA, USA). HEK293T, MCF7, U87, SW480, and Hep3B cells were cultured in high-glucose DMEM (Dulbecco’s Modified Eagle Medium, SH30022.01, ThermoFisher Scientific, Waltham, MA, USA) medium, HCT116 cells were cultured in McCoy’s 5a (16600108, ThermoFisher Scientific) medium, A549 cells were cultured in F12K medium (SH30026.01B, Hyclone, Logan, UT, USA), and HT29 and PANC-1 cells were cultured in modified RPMI medium (SH30809.01, Hyclone) with 10% bovine fetal serum (FBS, 10100147, ThermoFisher Scientific) and 1% penicillin/streptomycin at 37 °C in a humidified incubator containing 5% CO_2_**.**

### 4.3. Cell Viability Measurement

The effects of compounds on inhibiting CRC cell proliferation and the IC_50_ values of compound **6d** were measured by 3-(4,5-dimethyl-2-thiazolyl)-2,5-diphenyl-2-H-tetrazolium bromide (MTT, Beyotime, ST316, Shanghai, China) assay. Briefly, cancer cells were seeded on 96 well-plates at a density of 3 × 10^3^–6 × 10^3^ cells per well with 200 μL complete medium. Following that, CRC cells were treated with compounds at a concentration of 10 μM for 48 h after incubation for 24 h. For IC_50_ values measurement of compound **6d**, CRC cells were exposed to 0, 0.78, 1.56, 3.125, 6.25, 12.5, 25, 50, and 100 μM for 48 h after incubation for 24 h. After that, each well had 20 μL MTT (5 mg/mL) added and was incubated for another 4 h. The medium was then removed and the insoluble formazan inside cells was dissolved in 200 μL DMSO (Dimethylsulfoxide). The plate was placed into the table shaker and shaken for about 10 min at 140 rpm/min. The optical density (OD) of each well was measured by the microplate reader (Bio-Tek, Winooski, VT, USA) at 570 nm. The OD value of the control wells was used as 100%. IC_50_ values of all compounds were calculated by GraphPad Prism 9.0 (GraphPad Software, San Diego, CA, USA) and the experiments were repeated three times.

### 4.4. Flow Cytometry

Apoptosis induced by compound **6d** in HCT116, HT29, and SW480 cells was performed with flow cytometry using the Annexin V-FITC/PI kit (Becton Dickinson, San Jose, CA, USA). Briefly, CRC cells were harvested after treatment with compound **6d** for 8 h, and then washed three times with phosphate buffered saline (PBS) and resuspended with 1 × binding buffer to a cell density of 1 × 10^6^ cells/mL. Subsequently, cells were stained with 5 μL Annexin V-FITC and 5 μL PI (50 μg/mL) and incubated in the dark for 15 min. The stained cells were immediately detected using flow cytometry and analyzed with FlowJo 7.6 software (Becton Dickinson, San Jose, CA, USA).

### 4.5. Lentiviral Preparation and Viral Infection

The lentiviral Caspase 3-shRNA, Bcl-2-HA, Bcl-2-Flag, GFP-LC3B, or mcherry-EGFP-LC3B overexpression system were co-transfected with lentiviral packaging vectors pSPAX2 and pMD2G into HEK293T cells using Lipo8000™ Transfection Reagent (C0533, Beyotime), respectively. Virus particles were collected after 48 h infection, filtered through a 0.22 μm membrane, added to HCT116 cells, and then incubated for 24 h at 37 °C. The medium was replaced and HCT116 cells were selected for puromycin tolerance (5 μg/mL) to obtain Caspase 3 knock-down, GFP-LC3B, or mcherry-EGFP-LC3B overexpression cells.

### 4.6. Isolation of Cytosolic and Mitochondrial Fractions

The Cell Mitochondria Isolation Kit (C3601, Beyotime Biotechnology, Shanghai, China) was utilized to isolate the cytosolic and mitochondrial fractions. Briefly, HCT116 cells were collected using trypsin-EDTA solution and washed with cold PBS three times. Cell pellets were resuspended in 300 μL of Mitochondria Isolate Reagent, incubated the suspension on ice for 10–15 min, and then homogenized with a glass homogenizer for 10–30 times. The cell homogenate was centrifuged at 600× *g* for 10 min at 4 °C. The supernatant was transferred to another tube and spun at 11,000× *g* for 10 min at 4 °C to pellet mitochondria. The supernatant was a cytoplasmic protein without mitochondria. Both the mitochondrial pellet and supernatant were subjected to SDS-PAGE and western blotting.

### 4.7. Western Blotting (WB) and Immunoprecipitation (IP)

Cells were treated with compound **6d** for 4 h and lysed in cell lysis buffer (#9803, CST) containing phosphatase inhibitor cocktails (Roche, Mannheim, Germany) and protease inhibitor. The concentration of total proteins was measured using BCA (Bicinchoninic acid) kit (P0010, Beyotime). The cell lysate was subjected to sodium dodecyl sulfate polyacrylamide gel electrophoresis (SDS-PAGE) followed by WB analysis using indicated antibodies. Then, immunoreactivity was visualized using an odyssey two-color infrared fluorescence imaging system (LI-COR Biosciences, Lincoln, NE, USA).

For IP, the supernatant of whole-cell lysate was incubated with respective antibodies at 4 °C overnight. After incubation, the samples were further incubated with protein A + G agarose beads (P2012, Beyotime) for 2 h at 4 °C. The related beads were washed 5 times with 1 × IP buffer and then subjected to SDS-PAGE and WB.

### 4.8. Immunofluorescence Staining

1 × 10^5^ HCT116 cells treated with compound **6d** for 2 h and 4 h were seeded on coverslips and fixed in 4% paraformaldehyde for 10 min at 37 °C. Then, the cells were blocked in QuickBlock™ Blocking Buffer for Immunol Staining (P0260, Beyotime) for 1 h at 37 °C and incubated with corresponding antibodies at 4 °C overnight. After washing three times with sterile PBS, cells were stained with Alexa Fluor 488-conjugated anti-rabbit antibody (A-21206, 1:2000, ThermoFisher Scientific) or Alexa Fluor 594-conjugated anti-mouse antibody (A-11005, 1:2000, ThermoFisher Scientific) for 1 h at room temperature in cassette. Subsequently, cells were stained with 4,6-diamidino-2-phenylindole (DAPI, D1306, ThermoFisher Scientific) for 15 min at room temperature. Fluorescence images were captured and analyzed by High Content Analysis System (PerkinElmer, Waltham, MA, USA).

### 4.9. Time-Lapse Observation of Mcherry-EGFP-LC3B

HCT116 cells transfected with mcherry-EGFP-LC3B were seeded in a 96-well plate and treated with or without compound **6d**. The plate was cultured in High Content Analysis System-Operetta CLSTM, and images were captured at an interval of 1 h. To observe the dynamic variation of LC3B induced by compound **6d**, the process capturing these photos lasted 8 h in total.

### 4.10. In Vitro Cleavage Assay

Recombinant HDAC6 protein (31543, Active motif, Carlsbad, CA, USA) was incubated with or without 1 unit active caspase-3 (ab52101, Abcam) in a 25 µL reaction buffer containing 50 mM HEPES (pH 7.5), 3 mM EDTA, 150 mM NaCl, 0.005% Tween-20, and 10 mM DTT at 37 °C for 60 min. Finally, samples were treated with a loading buffer followed by WB analysis.

### 4.11. In Vitro Bax and Bcl-2 Interaction Assay

The recombinant His-BCL2 protein (Ag26118, Proteintech, Chicago, IL, USA) was pre-treated with Compound **6d** for 30 min at room temperature, and then incubated with recombinant His-Bax protein (ab173026, Abcam) in 1 × IP buffer at 37 °C for another 30 min. After incubation, the mixture was incubated with anti-Bcl2 antibody overnight at 4 °C and then the protein A + G agarose beads (P2012, Beyotime) were added at 4 °C for 6 h. Finally, the beads were washed with cold PBS and then analyzed by WB.

### 4.12. RNA Extraction and the HDAC6 Pre-mRNA Altered Splicing Assay

Total RNA from HCT116 cells treated with or without compound **6d** for 4 h and 8 h was extracted using Beyozol (R0011, Beyotime) according to the manufacturer’s instructions. The genomic sequence of HDAC6 spanning exons 26 to 29 was amplified using the forward primer 5′-GGGGGATCCGGGGCCTCAGAATCTCAG-3′ and the reverse primer 5′-GGGGCTCGAGAACAGCTTGTACTTTATT-3′. RT-PCR was performed using BeyoFast™ SYBR Green One-Step qRT-PCR Kit (D7268S, Beyotime) according to manufacturer’s instructions, and then the PCR products were assessed to detect the relative abundance of different splice forms by applying to 2% agarose gels. The results were analyzed using the Tanon 5200 Imaging System (Tanon Science & Technology Co., Ltd., Shanghai, China).

### 4.13. In Vivo Mouse Xenograft Model

All animal studies performed in the present study were reviewed and approved by the Ethics Committee for Animal Studies at Chongqing University of Arts and Sciences, Chongqing, China. Nude Balb/c mice were purchased from Hunan SJA Laboratory Animal Co., Ltd. All experimental procedures were performed while the animals were under 2.5% isoflurane gas anesthesia.

Nude Balb/c mice were purchased from Hunan SJA Laboratory Animal Co., LTD. (Hunan, China). During the assay, 1 × 10^6^ HCT116 cells were suspended in 100 μL serum-free McCoy’s 5a and inoculated subcutaneously on the flanks of 4–6-week-old female Nude Balb/c mice weighing 16–20 g. The mice were divided into 5 groups (n = 6 per group) and intraperitoneally administered every 3 days with vehicle, 3 mg/kg, 10 mg/kg, 30 mg/kg, or 100 mg/kg of compound **6d** dissolved in 100 μL solvent containing 5% DMSO, 30% PEG300, 10% Tween −80, and 55% saline when the mean tumor volume was approximately 100 mm^3^. Tumor length (L) and width (W) were measured using a vernier caliper every 3 days, and tumor volume was calculated according to the formula: V (volume) = (L × W2)/2. Mice were sacrificed after 32 days treatment, and the xenograft tumors were dissected and weighed. Subsequently, the tumor tissues were collected, fixed in 4% PFA and embedded with paraffin for Hematoxylin and eosin staining.

### 4.14. Statistical Analysis

All experiments were performed in triplicates. GraphPad Prism version 9.0 was used for statistical analysis. Data were presented as mean ± standard deviation, and the ANOVA (Analysis of variance) method was used to compare differences between groups. *p* < 0.05 was considered to be significant.

## Data Availability

Not applicable.

## Supplementary Materials

The supporting information can be downloaded at: https://www.mdpi.com/article/10.3390/ijms24076662/s1.
