# Supplementary material for: Pharmacological Targeting of Bcl-2 Induces Caspase 3-Mediated Cleavage of HDAC6 and Regulates the Autophagy Process in Colorectal Cancer
Source: Int J Mol Sci. 2023 Apr 3;24(7):6662. doi: 10.3390/ijms24076662 (PMC10095469; doi:10.3390/ijms24076662)
Supplement: Supplementary file 1 [file ijms-24-06662-s001.zip › ijms-2215026-supplementary.pdf]

Supplementary Information

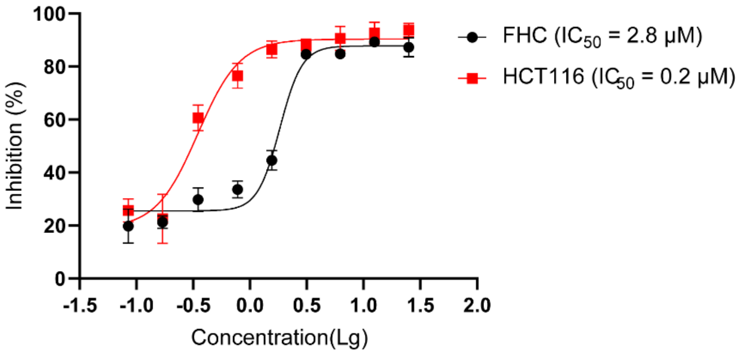

Figure S1. The IC_{50} values of Compound 6d in FHC and HCT116 cell lines

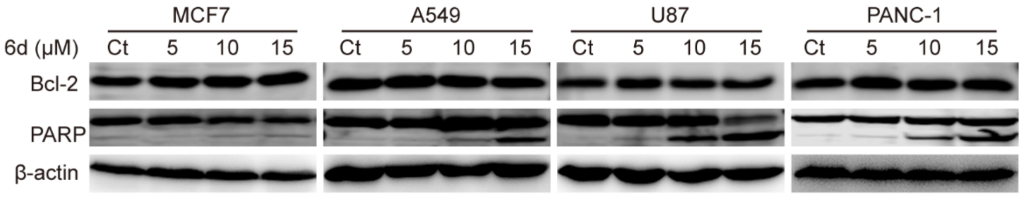

Figure S2. Compound 6d promotes PARP cleavage, but does not affects Bcl-2 expression in different cancer lines. MCF7 (breast cancer cell), A549 (human non-small cell lung cancer cell), U87 (glioma cell) and PNAC-1 (pancreatic caner cell) cells were treated with indicated concentrations of compound 6d for 8 h. Then, Bcl-2 and PARP expression levels were detected using corresponding antibodies. β-tubulin was loaded as a control.

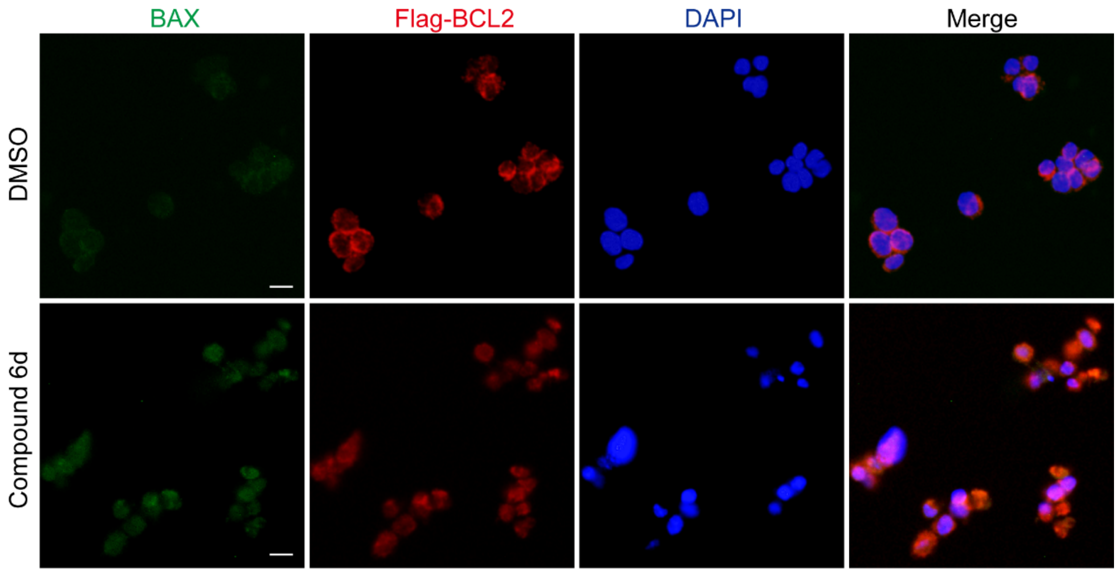

Figure S3. Exogenous Flag-Bcl-2 is capable to co-localize with Bax which localizes at the mitochondrial membrane. HCT116 cells were transfected with Flag-Bcl-2 and treated with or without 15 μM compound 6d for 4h. Cells were then washed, fixed with 4% paraformaldehyde, blocked with 5% BSA in PBS, and stained with anti-flag and anti-Bax antibodies. Images were captured with on a High Content Analysis System (PerkinElmer, Waltham, MA, USA). All images were equally adjusted for brightness and contrast using ImageJ (NIH, Bethesda, MD). Scale bar, 10 μm.

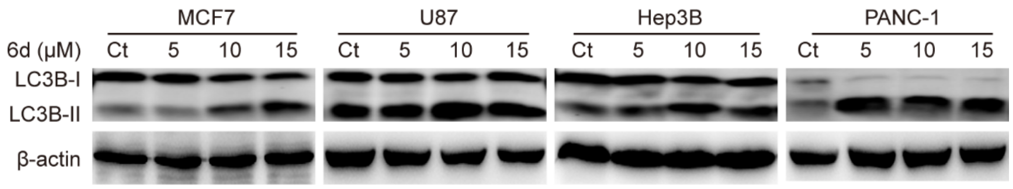

Figure S4. Compound 6d regulates autophagy in different cancer cell lines. MCF7, U87, Hep3B (hepatoma carcinoma cell) and PNAC-1 cells were treated with indicated concentrations of compound 6d for 8 h, and followed by western blotting with LC3 antibody.

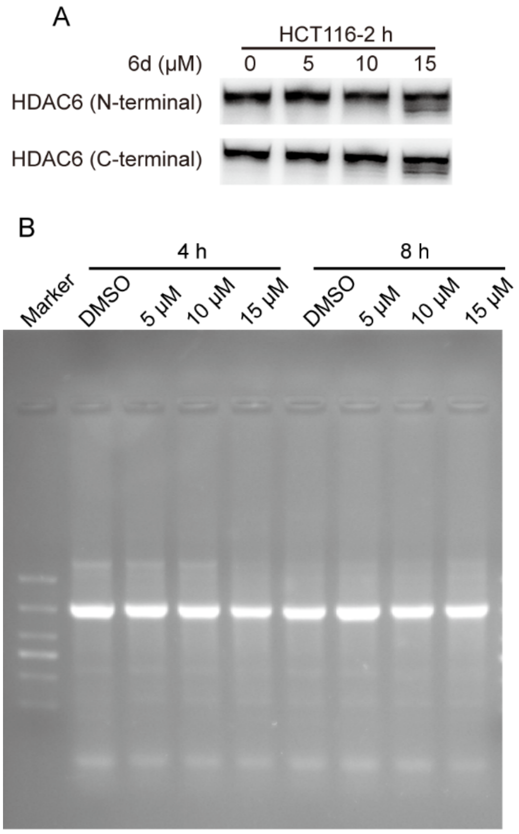

Figure S5. The induction of HDAC6 cleavage in compound 6d treated HCT116 cells occurs at the protein level rather than at the mRNA level. (A) Both the antibodies against N- or C-terminal of HDAC6 can recognize the cleaved fragments after treatment with indicated concentrations of compound 6d for 2 h. (B) Compound 6d treatment has no effect on transcript level of HDAC6. HCT116 cells were treated with indicated concentrations of compound 6d for 4 h and 8 h, respectively, and then the mRNA level was analyzed with qRT-PCR.

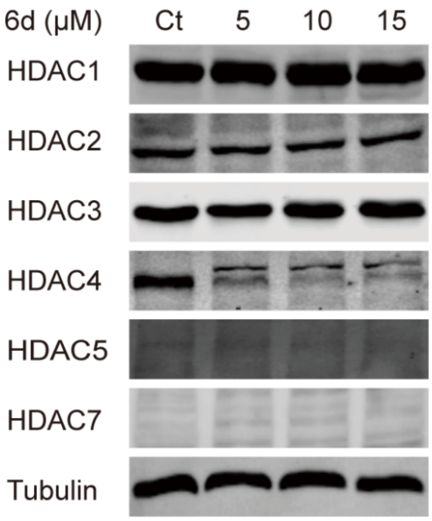

Figure S6. Compound 6d has no cleavage effect on other HDACs. HCT116 cells were treated with indicated concentrations of compound 6d, and further analyzed using western blotting with corresponding antibodies to detect the effect of this inhibitor on HDACs.
